# Polyphenol-rich black currant and cornelian cherry juices ameliorate metabolic syndrome induced by a high-fat high-fructose diet in *Wistar* rats

**DOI:** 10.1016/j.heliyon.2024.e27709

**Published:** 2024-03-11

**Authors:** Marija Paunovic, Maja Milosevic, Olivera Mitrovic-Ajtic, Natasa Velickovic, Bojana Micic, Olgica Nedic, Vanja Todorovic, Vesna Vucic, Snjezana Petrovic

**Affiliations:** a Group for Nutritional Biochemistry and Dietology, Centre of Research Excellence in Nutrition and Metabolism, Institute for Medical Research, National Institute of Republic of Serbia, University of Belgrade, 11000, Belgrade, Serbia; b Group for Neuroendocrinology, Institute for Medical Research, National Institute of Republic of Serbia, University of Belgrade, 11000, Belgrade, Serbia; c Group for Molecular Oncology, Institute for Medical Research, National Institute of Republic of Serbia, University of Belgrade, 11000, Belgrade, Serbia; d Department of Biochemistry, Institute for Biological Research “Siniša Stanković”, National Institute of the Republic of Serbia, University of Belgrade, 11060, Belgrade, Serbia; e Institute for the Application of Nuclear Energy (INEP), University of Belgrade, 11080, Belgrade, Serbia; f Department of Bromatology, University of Belgrade - Faculty of Pharmacy, 11221, Belgrade, Serbia

**Keywords:** Metabolic syndrome, Polyphenol-rich juices, Black currant, Cornelian cherry, High-fat high-fructose diet, Animal model

## Abstract

Diets high in fat and sugar lead to metabolic syndrome (MetS) and related chronic diseases. We investigated the effects of commercially available, cold-pressed polyphenol-rich black currant (BC) and cornelian cherry (CC) juices on the prevention of MetS in *Wistar* rats induced by a 10-weeks high-fat high-fructose (HFF) diet. Juice consumption, either BC or CC, with a HFF diet resulted in lower serum triglycerides compared to only the HFF consumption. Both juices also mitigated the effects of HFF on the liver, pancreas, and adipose tissue, by preserving liver and pancreas histomorphology and reducing visceral fat and adipocyte size. Furthermore, supplementation with both juices reduced glucagon and up-regulated insulin expression in the pancreas of the rats on the HFF diet, whereas the BC also showed improved glucose regulation. BC juice also reduced the expression of IL-6 and hepatic inflammation compared to the group only on HFF diet. Both juices, especially BC, could be a convenient solution for the prevention of MetS in humans.

## 1 Introduction

Metabolic syndrome (MetS) has become a global epidemic and health concern. Multiple factors have been identified as the cause of MetS, including obesity, a sedentary lifestyle, and improper nutrition [1]. Consumption of energy-dense foods rich in refined carbohydrates and fats, a hallmark of the modern Western-type diet, is strongly associated with increased development of MetS characteristics. These include abdominal obesity, dyslipidemia, hyperglycemia, and hypertension, leading to type 2 diabetes (T2DM), non-alcoholic fatty liver disease (NAFLD), and cardiovascular disease (CVD) [2].

Numerous studies from human and animal studies revealed positive effects of polyphenols on the reduction of MetS symptoms [[3], [4], [5], [6]]. The most investigated polyphenols are from aronia and pomegranate seeds and peel. We already demonstrated the benefits of those polyphenols on dyslipidemia, fatty acid profiles, and blood pressure [[7], [8], [9], [10]]. According to animal and human studies, polyphenols from berries also prevent the development of T2DM by regulating glucose homeostasis and insulin sensitivity [11] and reducing cardiovascular complications [12]. However, some other polyphenol-rich berry fruits with potentially high antioxidant, anti-inflammatory, and hypoglycemic and/or hypolipidemic effects are less investigated.

Among them, black currant (BC) berries contain many phenolic compounds potentially beneficial for human health [13]. In particular, BC is rich in anthocyanins, proanthocyanidins, quercetin, myricetin, isorhamnetin, and phenolic acids [[14], [15], [16]]. These compounds have been shown to have an inhibitory effect on the development of certain cancers, cardiovascular disorders, and inflammation-related conditions [14,[17], [18], [19]]. Similarly, cornelian cherry (CC) berries are also a rich source of polyphenols and other bioactive compounds [20], mostly anthocyanins and iridoids, whose pharmacological action has been proven for their antiatherogenic, anti-inflammatory, and neuroprotective properties [21]. Although these two fruits are well known in Serbian folk medicine [22,23], there is not enough scientific evidence to confirm their medicinal effects. Previous animal studies conducted on supplementation with different BC and CC products have shown favorable health effects [24,25]. Moreover, we recently conducted a study on the effects of BC and CC juices consumption on systemic oxidative stress and found that both juices, especially BC, had a protective effect on the maintenance of redox homeostasis in animals with HFF-induced MetS [26]. These antioxidative effects also suggested that both BC and CC can have beneficial effects on some other components of MetS, such as blood glucose and lipid levels, inflammation, and liver steatosis. Furthermore, cold-pressed juices are convenient and easily accessible throughout the year and thus can be an easy way to improve dietary habits in today's urban lifestyle and ameliorate some of the adverse effects of the Western-type diet, being commonly consumed, despite known to be pro-inflammatory..

Given the aforementioned beneficial effects of polyphenols and other bioactive compounds from BC and CC, we hypothesized that commercially available BC and CC juices might prevent and/or ameliorate metabolic and cardiovascular disturbances in the *Wistar* rat model of MetS induced by a high-fat high-fructose diet. Therefore, the aim of this study was to investigate the potential influence of commercially available BC and CC juices on selected biochemical, histological, and molecular markers of MetS, such as fat accumulation, blood lipids, insulin resistance, high blood pressure, increased inflammation, and others disorders, induced by an HFF diet in rats.

## 2 Materials and methods

### 2.1 Animals and experimental design

The experiments were carried out on 3.5-month-old male *Wistar* rats. The animals were kept under controlled conditions, 12 h light-dark cycle, 22 ± 2 °C, and had free access to food and liquid. All experimental procedures were done according to the National Law of Animal Welfare (“Official Gazette of RS” 41/09 and 39/10) and the Directive 2010/63/EU. The study protocol was approved by the Ethics Committee of the Institute for Medical Research, National Institute of Republic of Serbia, University of Belgrade, Serbia, and Veterinary Administration, Ministry of Agriculture, Forestry and Water Management, Republic of Serbia (No. 323-07-06069/2019-05), June 26, 2019, and in line with the ARRIVE protocol.

Rats were randomly divided into four groups (*n* = 9 each). The control group was placed on a standard chow diet (Agrofirm, Pozarevac, Serbia) and tap water, HFF group-was on a standard diet enriched with 25% sunflower oil, 20% fructose, and 0.1% cholic acid (HFF diet) and tap water. The BC group was on an HFF diet with 20% cold-pressed black currant juice in tap water (juice/water, 1:5, v/v), while the CC group was on an HFF diet with 20% cold-pressed cornelian cherry juice in tap water (juice/water, 1:5, v/v). The composition of the standard chow diet and HFF diet fed to rats is presented in Supplementary Table S1. Commercially available black currant (*Ribes nigrum* L.) and cornelian cherry (*Cornus mas* L.) juices were purchased from a local manufacturer. Juices were produced from cold-pressed fresh fruits and brief (a few seconds) pasteurization, with no additional sugars, vitamins, or water added. According to the manufacturer's declaration, BC juice contained 14 g of sugar, 1 g of fats, 0.3 g of proteins, and 63 kcal per 100 mL, while CC contained 12 g of sugar, 0.2 g of fats, 0.5 g of proteins, and 52 kcal per 100 mL of juice. The energy (caloric) value of the juices was included in the total daily energy count. The sugar from the juices did not significantly affect total sugar and energy intake, which included the energetic value of 20% BC or 20% CC juice (12.6 kcal/100 mL, and 10.4 kcal/100 mL, respectively) and their daily consumption per rat (26.1 mL, and 27.1 mL, respectively). BC juice contained 1.4 g GAE/L of total polyphenols, while CC contained 1.0 g GAE/L. The phenolic profile and the content of different phenol classes in juices were also determined and recently described in detail in our previous study [26].

After 10 weeks of treatment, the animals were placed on overnight fasting, and blood was collected via cardiac puncture after the animals were anesthetized with 4% isoflurane.

### 2.2 Food and liquid consumption, body mass, and adiposity assessment

The food and liquid intakes were recorded every week. The average intake is presented in Table 1. Body mass was measured once a week during the 10-weeks treatment and presented as the baseline mass before treatment and the final mass at the end of treatment. After the animals were sacrificed, total visceral fat was collected and weighed, and the adipose tissue mass was divided by the total weight of the rats. Body adiposity was expressed as a percentage of adipose tissue of total body mass.

**Table 1:** Body mass and body fat quantification, food and liquid intake.

	Control	HFF	BC	CC
Baseline body mass (g)	368 ± 24	371 ± 24	352 ± 26	368 ± 30
Final body mass (g)	401 ± 31	424 ± 19	391 ± 33	441 ± 51*†
Body fat (%)	3.28 ± 0.75	6.32 ± 1.27***	5.34 ± 0.83***#	6.89 ± 1.63***
Food intake (g/day/cage)	72.99 ± 10	49.78 ± 8***	43.37 ± 6***	41.8 ± 5***#
Water/juice intake (mL/day/cage)	84.7 ± 8.73	73.5 ± 15.22	78.3 ± 9.45	81.3 ± 7.10
Calorie intake (kcal/day/rat)	86.3 ± 5.4	83.3 ± 4.7	88.3 ± 6.1	83.4 ± 3.7

HFF- rats on a high-fat high-fructose (HFF) diet, BC- rats on an HFF diet + 20% black currant juice, CC- rats on an HFF diet + 20% cornelian cherry juice. 1 kcal = 4.2 kJ.

The data are presented as means ± SD, (n = 9, 3 rats per cage). *P < 0.05 *vs* Control, **P < 0.01 *vs* Control, ***P < 0.001 *vs* Control; #*P* ≤ 0.05 *vs* HFF; †*P* ≤ 0.05 *vs* BC group.

### 2.3 Biochemical analysis of plasma samples and glucose tolerance test

Plasma was separated by centrifugation at 3000×*g* at 4 °C for 15 min. The levels of triglyceride (TG), total cholesterol (TC), HDL-cholesterol (HDL-C), and LDL-cholesterol (LDL-C) were analyzed using Clinical chemistry analyzer (Cobas c111, Roche Diagnostics, Basel, Switzerland) and Roche Diagnostics kits, respectively, following the instructions of the manufacturer. Plasma insulin levels were measured using radioimmunoassay (RIA) (INEP, Belgrade, Serbia).

An intraperitoneal glucose tolerance test (IPGTT) was performed 3 days before the end of the treatment. Rats were fasted for 4 h when juices were temporarily replaced with water. A 25% glucose solution dissolved in sterile saline was injected intraperitoneally (2 g/kg) without anesthesia, to avoid the effect of an anesthetic on glucose level and kinetics of glucose disposal. Blood was obtained from the tail tip, and the glucose levels were monitored at baseline and 15, 30, 60, 90, and 120 min after glucose load, with a glucometer (Contour Plus, Bayer, Germany). The area under the glycemic curve (AUC) over the course of the experiment was calculated using the trapezoidal rule (AUC glucose 0–120 min, mmol/L *vs* the lowest value).

### 2.4 Blood pressure measurement

Blood pressure was recorded in conscious animals previously trained for adaptation to the method, by non-invasive tail-cuff method (Rat Tail Cuff Method Blood Pressure Systems (MRBP-R), IITC Life Science Inc. USA). Briefly, after 20 min in an incubator at 37 °C to dilate the caudal artery, animals were individually restrained in a clear acrylic restrainer, the room temperature was maintained at 23 °C for accurate blood pressure measurements, and at least four measurements for each parameter were recorded to obtain a mean result.

### 2.5 Histological studies

Samples of liver, pancreas, and visceral adipose tissue (pooled depots of retroperitoneal and perirenal white adipose tissue) were fixed in 4% paraformaldehyde for 24 h, washed in water, dehydrated in ethanol gradient, cleared in xylene, and embedded in paraffin. The 5 μm thick tissue sections were cut on a Leica RM2065 microtome, stained with hematoxylin and eosin**,** and mounted in Canada balsam. Images were examined under Olympus AX70 light microscope (Hamburg, Germany) with an objective magnification of ×20 or ×40 and recorded using a high-resolution digital camera (Olympus DP50, Tokyo, Japan). The pancreas immunohistochemical staining for glucagon and insulin was performed with a monoclonal antibody against glucagon (1:100 overnight at 4 °C, Ab10988, Abcam) and a guinea pig polyclonal antibody against insulin (1:100 overnight at 4 °C, Ab7842, Abcam), respectively. After a brief wash in PBS, immunostaining was performed using the streptavidin–biotin technique and DAB Substrate/Chromogen System for visualization (Novocastra Peroxidase Detection System kit, Leica Biosystems, Wetzlar, Germany). Control sections without the primary antibody were processed in parallel. The nuclei were counterstained with Mayer's hematoxylin. Three fields of view were analyzed by measuring the diameter (μm) of adipose cells in every tissue sample. The average cell diameter was calculated for each experimental group. Semiquantitative analysis of glucagon and insulin expression was performed by determining the intensity of staining (0- none, 1 - weak, 2 – moderate, 3 - intensive) and the number of immunoreactive cells (1 - less than 50%, from 51 to 100%).

### 2.6 RNA isolation and real-time polymerase chain reaction (RT-PCR)

Total RNA was isolated from liver tissue using TRIzol® Reagent (AmBion, Life Technologies, Carlsbad, CA, USA) according to the manufacturer's instructions. Quantitative and qualitative evaluation of the isolated RNA was performed spectrophotometrically (OD 260/280 > 1.8 was considered satisfactory) and on 2% agarose gel. Reverse transcription was performed using a high-capacity cDNA Reverse Transcription Kit (Applied Biosystems, Foster City, CA, USA) according to the manufacturer's instructions. The cDNAs were stored at − 70 °C until use.

Quantification of TNFα, IL-1β, and IL-6 gene expression in the liver was performed by TaqMan® real-time polymerase chain reaction (PCR). The following FAM-labeled probe sets were used: TNFα (Rn01525859_g1), IL1β (Rn00580432_g1) and IL6 (Rn01410330_m1). Quantitative normalization of cDNA in each sample was performed using TBP (Rn01455646_m1∗) as endogenous control, all obtained from Applied Biosystems Assay-on Demand Gene Expression Products. Real-time PCR was performed using QuantStudio™ Real-Time PCR Systems (Applied Biosystems, Foster City, CA, USA) as previously published (Vasiljevic et al., 2013). Relative quantification of gene expression was examined using the comparative 2^−ΔΔCt^ method described by Livak and Schmittgen [27]. The results were analyzed by QuantStudio™ Design and Analysis software v1.3.1 (Applied Biosystems, Foster City, CA, USA) with a confidence level of 95% (*P* ≤ 0.05).

### 2.7 Statistical analysis

Results are shown as means ± SD. Parameters with normal distribution were analyzed with ANOVA followed by Tukey's post-hoc test for the differences in subgroups, while asymmetrically distributed variables were analyzed by the Mann-Whitney and Kruskal-Wallis tests. The differences were considered significant at *P* ≤ 0.05.

## 3 Results

### 3.1 Body mass, adiposity, food, and water/juice intake quantification

Baseline body masses were similar in all study groups, while at the end of the treatment, the CC group had significantly higher body mass than the control and BC-treated groups (Table 1). However, a prolonged energy-rich diet resulted in a significantly higher (*P* < 0.001) proportion of adipose tissue in all HFF groups when compared with usual diet-fed control rats. Still, the proportion of adipose tissue in BC-treated rats was significantly lower (*P* < 0.05) compared to the HFF group (Table 1). The rats on HFF significantly reduced daily food intake in comparison with the control group. The rats on juice supplementation also slightly reduced food intake (see Table 1). There was no significant difference in daily liquid consumption and total energy intake among the experimental groups.

### 3.2 Biochemical analysis of plasma samples

The HFF diet did not alter the plasma lipid levels, except that the HDL-C level was lower compared to the control (Table 2). On the other hand, both juice-supplemented groups had significantly lower plasma triglyceride levels, as compared to either control or HFF groups.

**Table 2:** Effects of HFF diet and juices supplementation on plasma lipid status, baseline glucose, insulin concentrations, and IPGTT test.

	Control	HFF	BC	CC
Total cholesterol (mmol/L)	1.56 ± 0.23	1.53 ± 0.20	1.23 ± 0.23**#	1.30 ± 0.14**
HDL-C (mmol/L)	0.98 ± 0.13	0.83 ± 0.16*	0.62 ± 0.12***#	0.66 ± 0.14***#
LDL-C (mmol/L)	0.18 ± 0.12	0.32 ± 0.15	0.38 ± 0.16*	0.35 ± 0.17
TG (mmol/L)	0.85 ± 0.10	0.83 ± 0.29	0.57 ± 0.07***##	0.61 ± 0.07***##
Glc (mmol/L)	5.22 ± 0.33	5.56 ± 0.49	5.75 ± 0.97	5.20 ± 0.40
Insulin (IU/mL)	28.8 ± 5.17	28.1 ± 6.46	35.3 ± 10.31	38.9 ± 8.30
IPGTT glucose peak_30 min_ (mmol/L)	8.35 ± 0.31	11.54 ± 1.12	10.16 ± 0.84	11.86 ± 1.61
IPGTT glucose AUC	870 ± 11.32	1276 ± 98.7**	1022 ± 22.8†	1105 ± 74.8

HFF- rats on a high-fat high-fructose (HFF) diet, BC- rats on an HFF diet and 20% black currant juice, CC- rats on an HFF diet, and 20% cornelian cherry juice. HDL-C- high-density lipoprotein cholesterol, LDL-C- low-density lipoprotein cholesterol, TG-triglycerides, Glc - glucose, IPGTT-intraperitoneal glucose tolerance test, AUC- area under the curve.

The data are presented as means ± SD (n = 9). *P < 0.05 *vs* Control, **P < 0.01 *vs* Control, ***P < 0.001 *vs* Control; #*P* ≤ 0.05 *vs* HFF group, ##*P* ≤ 0.01 *vs* HFF group, †*P* = 0.056 *vs* HFF group.

**Fig. 1:**
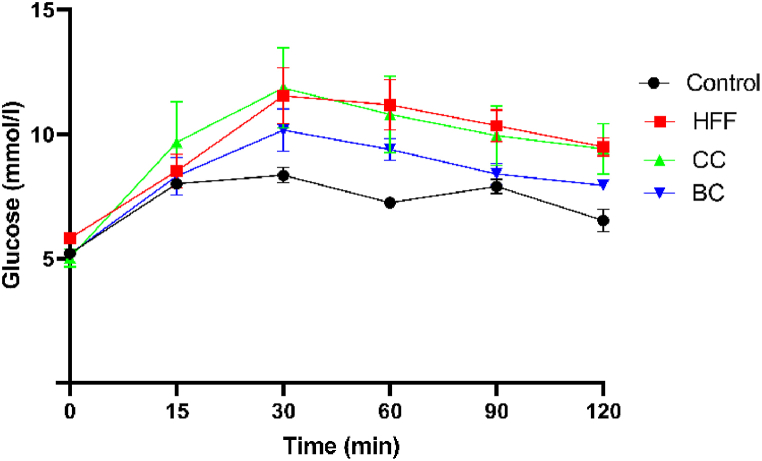
Intraperitoneal glucose tolerance test. HFF- rats on a high-fat high-fructose (HFF) diet, BC- rats on an HFF diet + 20% black currant juice, CC- rats on an HFF diet + 20% cornelian cherry juice. Values are expressed as means ± SEM (n = 9).

Fasting plasma glucose and insulin levels were similar in all experimental groups, as well as IPGTT glucose peaks (Table 2). Nevertheless, the area under the curve (AUC) was the highest in the HFF group and significantly higher compared to the control group (1276 ± 98.7 *vs* 870 ± 11.32, *P* < 0.01), indicating the development of glucose intolerance in HFF animals (Table 2). On the other hand, in BC- supplemented group AUC was lower compared to the HFF group only (P = 0.056). IPGTT showed non-significant differences in the glucose level at the different time points between groups (Fig. 1).

### 3.3 Blood pressure measurement

According to our results, the HFF diet significantly increased *(P* < 0.05) systolic blood pressure in all the HFF-fed groups independent of juice consumption (Table 3).

**Table 3:** Results of systolic and diastolic blood pressure at the end of the study.

	Control	HFF	BC	CC
SBP (mmHg)	128 ± 8	151 ± 10*	145 ± 10*	147 ± 11*
DBP (mmHg)	78 ± 9	87 ± 11	87 ± 8	87 ± 10

HFF- rats on a high-fat high-fructose (HFF) diet, BC- rats on an HFF diet + 20% black currant juice, CC- rats on an HFF diet + 20% cornelian cherry juice. SBP- systolic blood pressure, DBP- diastolic blood pressure. The data are presented as means ± SD (n = 9). *P < 0.05 *vs* Control.

### 3.4 Histopathological analysis of hepatic, adipose, and pancreatic tissue

**Fig. 2:**
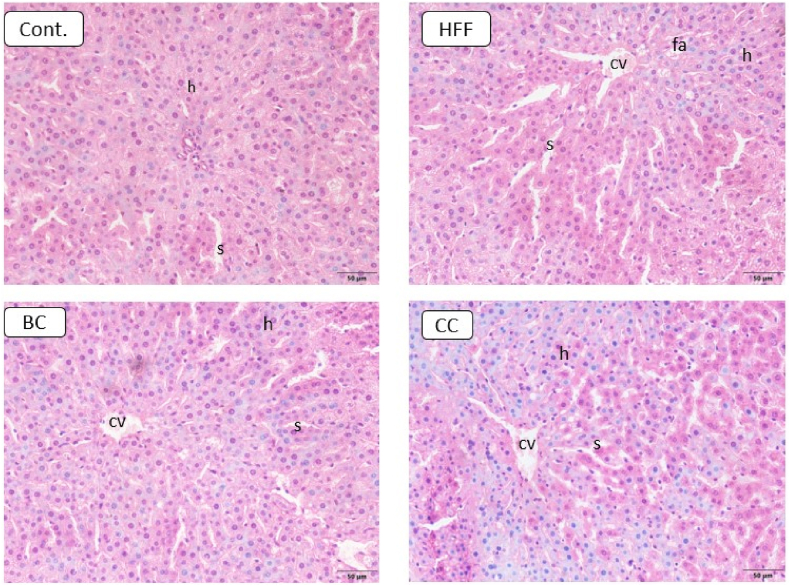
Changes in liver histology after treatment with different types of diet. Cont.- Control group, HFF- rats on a high-fat high-fructose (HFF) diet, BC- rats on HFF diet + 20% black currant juice, CC- rats on HFF diet + 20% cornelian cherry juice. (cv-central vein, h-hepatocytes, s-sinusoid, fa-fat accumulation). Magnification ×20.

In the liver of the HFF animals, vacuolar degeneration, fat accumulation, as well as visible changes in histological structures and architecture of certain zones were noted (Fig. 2). Hepatocytes had a trabecular arrangement and sinusoidal capillaries were notable. The changes were visible, especially in the structure of hepatocytes, which had irregular shapes and were separated with accumulated adipocytes. Capillaries were prominent with a large number of irregularly shaped erythrocytes. On the contrary, well-defined lobular liver structures were noticeable in BC and CC groups. The central veins were clearly visible in the center of the lobule, while inter-lobular portal spaces were localized at the periphery. Binuclear and mononuclear hepatocytes were visible in the samples. There was a normal trabecular arrangement of hepatocytes with sinusoidal capillaries covered with smooth endothelial and Kupffer cells, which had ovoid nuclei and transparent nucleoplasm. The blood vessels were histologically unchanged. Thus, in BC and CC groups the liver's structure was preserved, in line with control.

**Fig. 3:**
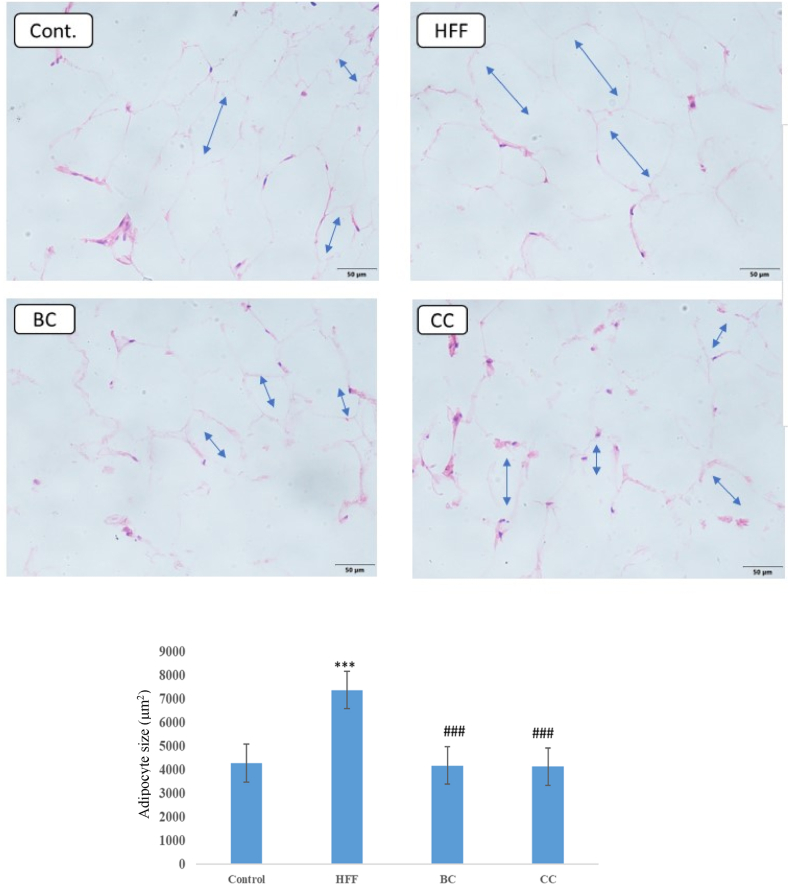
Influence of modified diet on adipose tissue morphology. Cont.- Control group, HFF- rats on a high-fat high-fructose (HFF) diet, BC- rats on HFF diet + 20% black currant juice, CC- rats on HFF diet + 20% cornelian cherry juice. (blue arrow indicates adipocyte lumen). ***P < 0.001 *vs* control group, ^###^*P* < 0.001 *vs* HFF group. Magnification ×20.

Histological analysis of rat's adipose tissue showed an increase in the size of adipocytes in the HFF group (7400 ± 210 μm^2^) in comparison to all other three groups including control (4300 ± 110 μm^2^), BC (4200 ± 120 μm^2^), and CC (4154 ± 190 μm^2^). On average, the diameter of adipocytes in HFF animals was almost twice as high as the diameter of adipocytes in the BC and CC groups. A similar trend was noted with adipose volume. It was also observed that BC and CC juice consumption led to cell structure preservation, which was not the case in rats that consumed the HFF diet alone (Fig. 3).

**Fig. 4:**
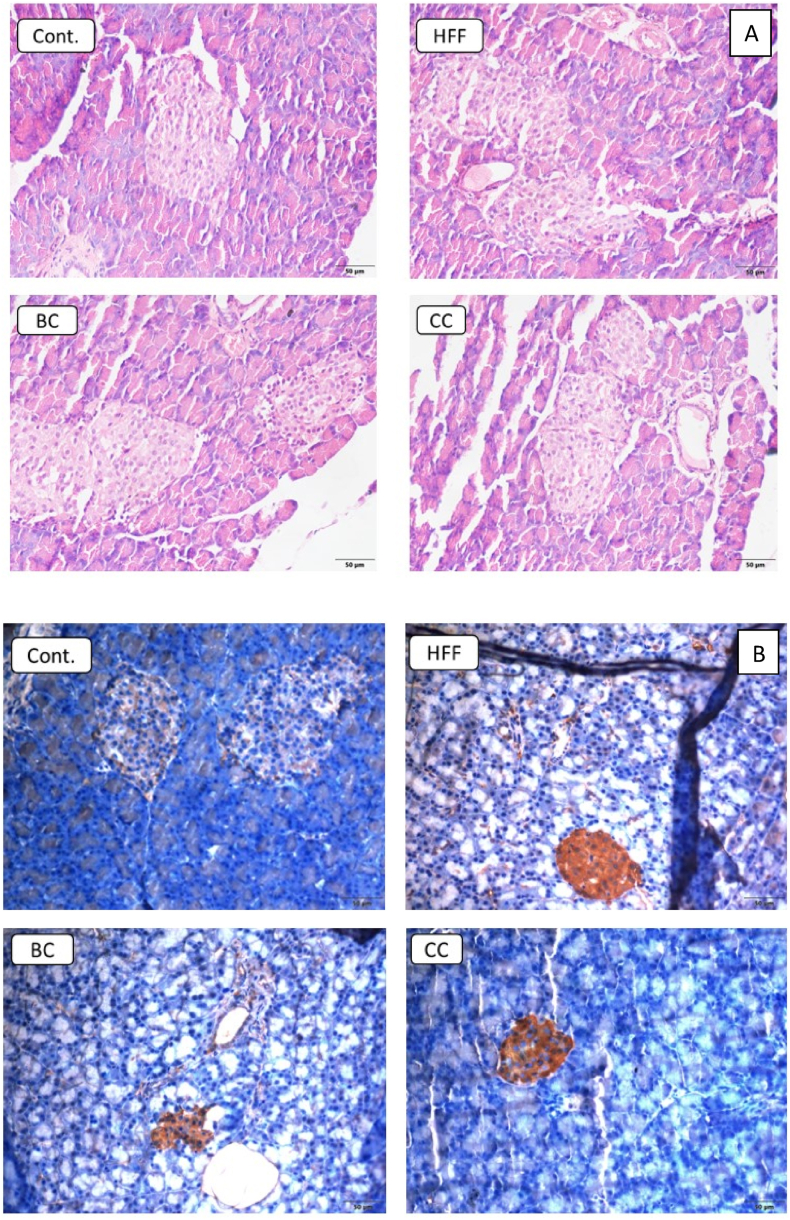

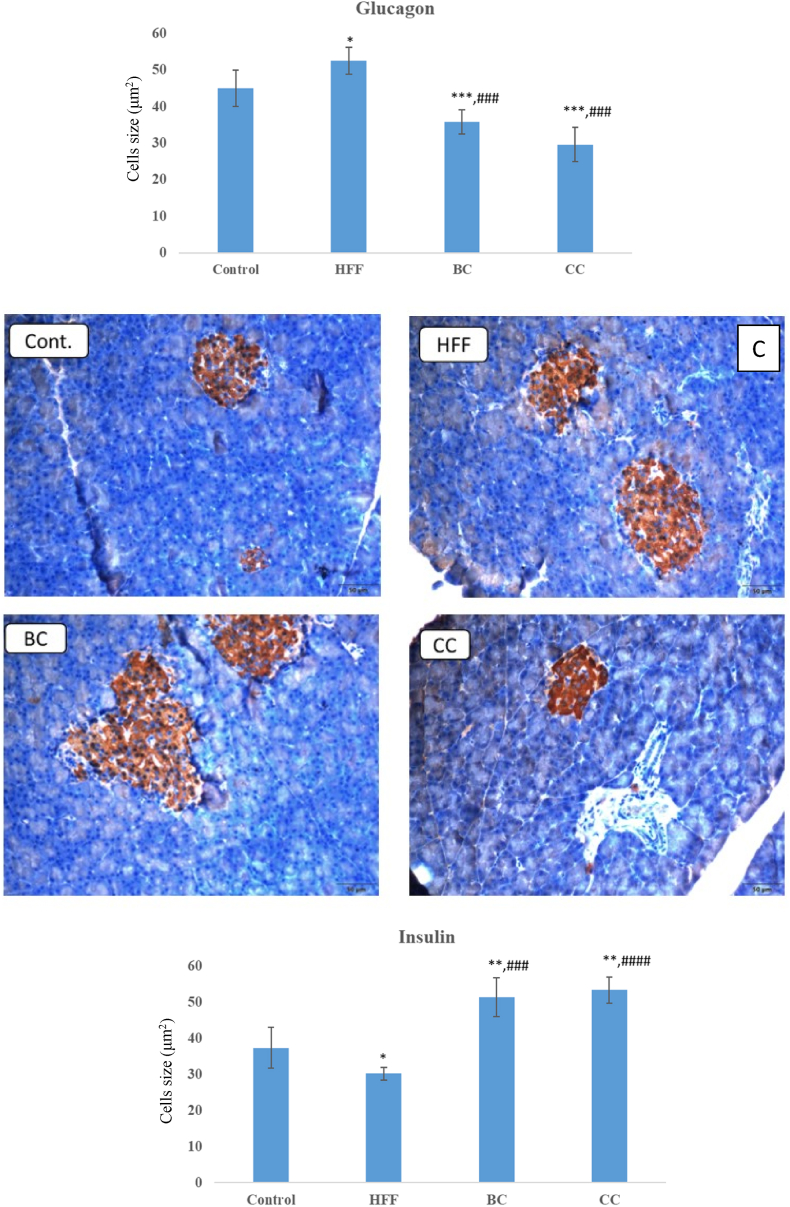
A) Morphology of islet of Langerhans. B) Immunohistochemical staining of glucagon immunoreactive cells in the islet of Langerhans. Magnification ×20. C) Immunohistochemical staining of insulin immunoreactive cells in the islet of Langerhans. Cont.- Control group, HFF- rats on a high-fat high-fructose (HFF) diet, BC- rats on HFF diet + 20% black currant juice, CC- rats on HFF diet + 20% cornelian cherry juice. *P < 0.05, **P < 0.01, ***P < 0.001 *vs* control group; ^###^*P* < 0.001 vs HFF group. Magnification ×20.

Furthermore, we detected pathological changes in the pancreatic tissue of HFF rats, represented by large and vacuolated acinar cells, and the degenerated islet of Langerhans with a marked decrease of β-cells (Fig. 4A). Again, the protective effect of CC and BC juice has been found, given that both supplemented groups had well-preserved islet of Langerhans and β-cells, as well as acinar cell and intra- and interlobular septa. In particular, an increased size of pancreatic islets was observed in the pancreas of BC rats.

In line with disturbed histomorphology, immunohistochemical analyses of pancreatic tissue (Fig. 4B) showed an increased number of glucagon immunoreactive cells in HFF rats (52.6 ± 3.6) compared to normal-fed control (45 ± 5.7). Again, supplementation with BC (35.8 ± 3.3) or CC (29.6 ± 4.7) juice had a beneficial effect by reducing glucagon expression in the pancreas of HFF diet-challenged rats. In addition, the BC (51.4 ± 5.4 number of insulin immunoreactive cells) and CC (53.4 ± 3.6) groups showed increased insulin expression in comparison with the HFF (30.2 ± 1.7) group (Fig. 4C).

### 3.5 Inflammatory cytokines and mRNA expression

**Fig. 5:**
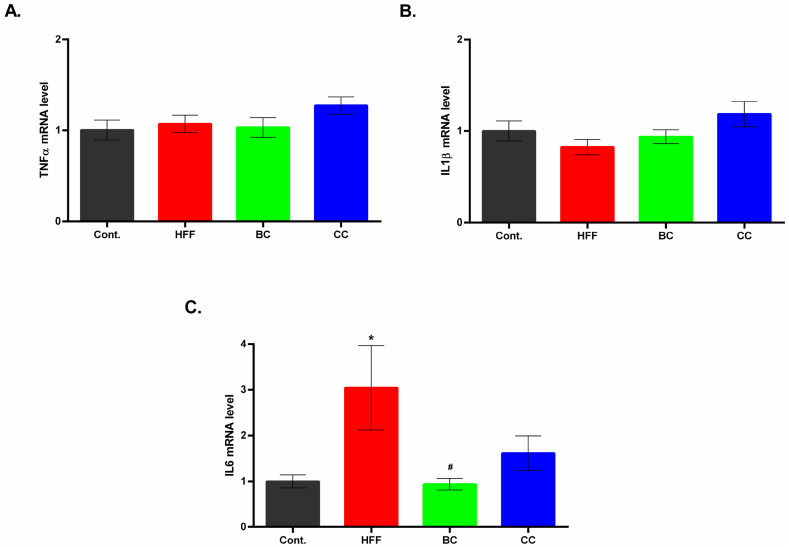
Relative quantification of A) TNFα, B) IL-1β, and C) IL-6 mRNA in the liver after ten weeks of high-fat high-fructose HFF diet, and BC and CC juice supplementation. The gene expression was normalized to β-actin gene expression. Cont.- Control group, HFF- rats on a high-fat high-fructose (HFF) diet, BC- rats on HFF diet + 20% black currant juice, CC- rats on HFF diet + 20% cornelian cherry juice. Values are expressed as means ± SEM (n = 9); *P < 0.05 *vs* control group; #*P* < 0.05 *vs* HFF group.

The expression of genes for inflammatory cytokines TNFα, IL-1β, and IL-6 in the liver is presented in Fig. 5(A–C). The IL-6 gene expression demonstrated a significant difference between groups (*p* = 0.03). The mRNA expression for IL-6 was significantly higher in the HFF group compared to the control group (*P* < 0.05), indicating an inflammatory effect of the HFF diet (Fig. 5C). At the same time, BC supplementation showed anti-inflammatory potential, as the expression of IL-6 mRNA was significantly lower in the BC group compared to the HFF animals (*P* < 0.05). Supplementation with CC exerted a similar effect, but it failed to reach statistical significance. No significant differences were observed in mRNA expression of the other two inflammatory cytokines, TNFα and IL-1β, between the groups (Fig. 5A and B).

## 4 Discussion

Although studies have shown that BC and CC juices may have hypolipidemic, hypoglycemic, and anti-inflammatory effects [25,28], their potential to prevent dietary-induced MetS has not been sufficiently investigated. In this study, we demonstrated the ameliorative effect of BC and CC juice on several MetS manifestations, including adiposity, glucose tolerance, hypertriglyceridemia, liver inflammation, and histopathological changes in the liver, adipose tissue and pancreas, induced by the HFF diet in a *Wistar* rats model.

In line with the reported literature, we found that food intake in all groups fed with the HFF diet was lower compared to the group on the standard chow diet [29]. This could be explained by a high-energy density of the HFF diet which improves satiety and reduces food consumption [30]. Therefore, no difference in body mass between HFF and standard chow-fed animals was found. Nevertheless, the differences were significant in adipose tissue mass, since a nearly doubled amount of adipose tissue was found in all HFFfed groups. Furthermore, results from our study revealed that co-administration of BC juice significantly reduced HFF diet-induced adiposity, while CC juice did not exert a statistically significant effect, despite a similar trend. The difference in the action of the juices may originate from variations in the overall concentration of polyphenols, as well as from differences in the presence of specific classes. Estimated from the total polyphenol content and the amount of juice consumed, the approximate amount of polyphenols that the animals were given in our study was 20 mg/kg body weight daily. This was the same dose as in our recently published paper Paunovic et al. [26], and similar to previously observed functional effects of these polyphenols [31,32]. When it comes to humans, equivalent doses would be reached by consumption of approximately one bottle, 200–250 mL, of juice daily. Dietary guidelines for the US and some European countries support moderate consumption of 100% fruit juice (75–224 mL daily) to improve nutrient intake and diet quality, while no association with an increase in the risk of obesity, type 2 diabetes, cardiovascular disease or poor glycemic control has been found [33,34]. Moreover, BC and CC juices are a good source of bioactive polyphenols, which are, in their own right, linked with health benefits. Thus, despite some sugar content also present, examined juices appear to offer more benefit than risk in attempt to prevent development of MetS. However, for implication in humans more studies may be needed.

In the BC juice, we also found that the concentration of flavon-3-ols (TF3C) was more than five times higher than in CC juice. Of note are the findings from some studies that demonstrated that TF3C influences lipolysis and reduces adipose tissue [35]. Previously published data on other polyphenol-rich juices also reported beneficial effects on rats’ adiposity [36]. There are several mechanisms of polyphenols' involvement in the management of adiposity/obesity, including inhibition of lipid and saccharide absorption, prevention of adipocyte differentiation and proliferation, and activation of AMP-activated protein kinase (that attenuates lipogenesis and enhance lipolysis) [37]. Polyphenols may affect adiposity and blood lipids through the downregulation of sterol regulatory element-binding protein 1c (SREBP-1c), which is an important transcription factor that regulates genes involved in FA synthesis and TG metabolism. This action results in the reduction of *de novo* lipogenesis while concurrently up-regulating peroxisome proliferator-activated receptor alpha (PPARα), leading to an increase in β-fatty acid oxidation [38,39]. Here we detected a significant reduction in adipose tissue mass, as well as in the size and number of adipocytes in the BC-supplemented rats compared to the HFF group. Since visceral adiposity is one of the main components of MetS, the obtained results can be considered significant to recommend the use of BC juice for the prevention of this widespread condition.

Dyslipidemia is another common feature of MetS, and it has been well-documented that prolonged consumption of fat and/or fructose leads to elevated levels of blood lipids [40]. An important finding of our study is that supplementation with BC or CC juices significantly reduced plasma TG levels even during prolonged HFF feeding in rats. The pathway of lipid metabolism modulation by polyphenols from natural sources is still not fully elucidated. Studies *in vitro* showed an inhibitory effect on enzymes involved in lipogenesis (fatty acid synthase, FAS) and impairment of aerobic energy metabolism by PPAR*g*, as well as increased expression of carnitine palmitoyltransferase I (CPT1A), the key enzyme of fatty acids β-oxidation [[41], [42]]. Both juices decreased the levels of HDL-C, which could be interpreted as a generally negative result concerning HDL-C's role in cholesterol and triglycerides transport in bloodstream. Noteworthy, cholesterol fractions in rats' blood differ from those in humans. For example, in humans, HDL-C carries about a quarter of the total amount of cholesterol in the blood, while in rats, HDL-C is a predominant cholesterol fraction [43]. Therefore, despite the observed decrease, it could be possibly expected that the beneficial physiological role of HDL-C in our juice-supplemented rats was preserved.

Many studies have linked high-fat, high-fructose diets with the development of glucose intolerance and insulin resistance [44,45]. Although our results did not reveal significant alternations in fasting blood glucose and insulin levels (in contrast to some other studies [46,47]), we found that the HFF diet increased glucose excursion during the IPGTT. To our knowledge, this is the first study that demonstrated decreased systemic insulin sensitivity and glucose intolerance after the HFF + cholic acid diet. The observed glucose intolerance most likely originated from enlarged visceral adipose tissue in HFF animals. Possible mechanisms by which hypertrophied adipocytes contribute to impaired insulin sensitivity include altered GLUT4 trafficking [48] and increased production and secretion of proinflammatory cytokines and adipokines. Additionally, due to the liver exposure to lipotoxic and proinflammatory metabolites from enlarged visceral adipose tissue, there is an increase in ectopic lipid accumulation and inflammation, contributing to decreased systemic insulin sensitivity [49]. This assumption is supported by our results demonstrating hepatic steatosis and increased expression of proinflammatory cytokines (e.g., IL-6) leading to impaired glucose tolerance in the HFF group. Polyphenol-rich natural products can also have a positive effect on blood glucose management since they augment glucose transport via GLUT4, β-cell function, insulin secretion, as well as AMPK-mediated suppression of hepatic gluconeogenesis [50,51]. A noteworthy finding of the current study is that BC juice decreased glucose excursion, which strongly suggests that BC juice consumption might be useful in preventing the development of insulin resistance in the MetS [52].

Our results showed that the HFF diet significantly increased systolic blood pressure in all HFF-fed groups. As shown previously, both excess fats and fructose in the diet lead to an increase in blood pressure via several mechanisms including increased salt retention, endothelial dysfunction, and overstimulation of the sympathetic nervous system [53,54]. On the contrary, some constituents previously identified in BC and CC juices, such as tartaric acid, malic acid, gallic acid, protocatechuic acid, and epigallocatechin [26] are known for their antihypertensive effects [55]. However, it is possible that the concentration in commercial juices used in our study was not sufficient to produce the same effects.

The effect of BC and CC juices in the management of dyslipidemia, particularly hypertriglyceridemia, corresponds to their effect on liver and adipose tissue morphology in HFF-fed animals. Alternations observed in the livers of our HFF rats include hepatocyte damage, fat accumulation, activated Kupffer cells, and lobular inflammation, all known to promote the development of NAFLD. Since both juices attenuated hepatocellular impairment and excessive lipid droplet accumulation induced by the HFF diet, their protective effects against NAFLD could be speculated. Our findings are in line with previous studies in humans and animals which found improvement in liver steatosis and NAFLD by polyphenol-rich supplements [56,57]. The liver steatosis, as detected in our HFF rats, is commonly associated with hepatic inflammation, characterized by infiltration of monocyte-derived macrophages and activation of resident Kupffer cells, as well as increased proinflammatory and decreased anti-inflammatory cytokines [58]. Both high-fat diets and high-fructose diets have been shown to promote liver steatosis and inflammation, with the combination of these diets promoting intrahepatic inflammation and hepatocellular injury to an even greater extent [59]. However, sequestration of reactive oxygen species (ROS), and thereby reduction of oxidative stress, could be another potential mechanism by which polyphenols may interrupt the cascade leading to steatosis and liver necrosis [60,61].

In our study, gene expression of proinflammatory cytokines TNFα nor IL-1β was not changed with any treatment, however, the IL-6 mRNA expression was significantly increased in HFF rats. The trigger for increased IL-6 gene expression could be due to the HFF diet-induced dysbiosis, which stimulates the release of gut-derived endotoxin-like lipopolysaccharide (LPS) and activation of resident Kupffer cells, resulting in a TNFα-dependent regulation of IL-6 in the liver [62]. IL-6 is a key factor in inducing acute phase response proteins and plays a central role in restoring normal hepatic function after liver injury. Therefore, the increase of IL-6 could be part of the adaptive response of the liver to HFF-mediated hepatocellular damage. Other studies also reported elevation of IL-6, but not TNFα and IL-1β, as a compensatory mechanism that prevents the development of hepatic steatosis at the early stage of NAFLD [63]. However, the sustained activation of IL-6 in the liver leads to a regulation of suppressors of cytokine signaling 3 (SOCS3), which, in turn, impairs insulin-mediated signaling in the liver and decreases both peripheral and systemic insulin sensitivity [64]. In the current study, BC juice decreased the expression of IL-6 mRNA and restored glucose regulation, indicating an anti-inflammatory effect in the liver and a possible role in the prevention of steatosis and glucose intolerance induced by the HFF diet.

Polyphenols from natural sources exert a beneficial activity for the prevention and treatment of diabetes [65]. They have a cytoprotective effect on pancreatic β-cells by activating the anti-apoptotic and inhibiting the pro-apoptotic signaling pathways, as well as by increasing cell resistance against the oxidative insult [26,66]. The results obtained herein indicate the efficiency of polyphenol-rich BC and CC juices against the histopathological changes and the loss of pancreatic β-cells during MetS development in rats. Moreover, both juices prevented an increase of glucagon immunoreactive cells and glucagon expression observed in the pancreas of HFF-fed animals without supplementation. Glucagon is a hormone produced by pancreatic α-cells, directly responsible for an increase in glucose levels in the bloodstream. Glucagon overexpression due to α-cells dysfunction is a common feature of diabetes [67]. In addition, the BC and CC groups had increased insulin expression compared to the HFF group, indicating the protective effect of juice supplementation. Our results show that BC juice supplementation ameliorates adverse MetS-related alternations in pancreatic structure and function.

In conclusion, our data show that BC juice may be useful in preventing some alterations associated with MetS development induced by the HFF diet in rats. The ameliorative effects of BC juice included reduction of adipose tissue, regulation of plasma TG, improvement of glucose tolerance, decreased glucagon and increased insulin expression, preservation of histomorphology in liver, adipose and pancreatic tissues, as well as suppression of hepatic inflammation. The beneficial effects of CC juice were mostly limited to glucagon and insulin expression, and protection of tissue morphology. These findings also suggest that consumption of BC juice, and to a lesser extent CC juice, may be useful for the prevention of MetS development and progression, as well as to counter the harmful effects of the Western-type diet. Future large human intervention studies are warranted to confirm the potential of the examined juices.

## Ethics statement

All experimental procedures were done according to the National Law of Animal Welfare (“Official Gazette of RS” 41/09 and 39/10) and the Directive 2010/63/EU. The study protocol was approved by the Ethics Committee of the Institute for Medical Research, National Institute of Republic of Serbia, University of Belgrade, Serbia, and Veterinary Administration, Ministry of Agriculture, Forestry and Water Management, Republic of Serbia (No. 323-07-06069/2019-05), June 26, 2019, and in line with the ARRIVE protocol.

## Funding statement

This work was supported by the Ministry of Science, Technological Development, and Innovation of the Republic of Serbia (No. 451-03-66/2024-03/200015 and 451-03-66/2024-03/200007).

## Data availability statement

Data included in article/supplementary material/referenced in article. The datasets presented in the article are not readily available as current investigations are still ongoing but will be made available upon reasonable request. Requests to access the dataset should be direct to marija.paunovic@imi.bg.ac.rs.

## CRediT authorship contribution statement

**Marija Paunovic:** Writing – original draft, Visualization, Methodology, Formal analysis, Data curation, Conceptualization. **Maja Milosevic:** Methodology, Formal analysis. **Olivera Mitrovic-Ajtic:** Writing – original draft, Formal analysis. **Natasa Velickovic:** Writing – original draft, Formal analysis. **Bojana Micic:** Formal analysis. **Olgica Nedic:** Formal analysis. **Vanja Todorovic:** Formal analysis. **Vesna Vucic:** Supervision, Conceptualization. **Snjezana Petrovic:** Writing – original draft, Supervision, Methodology, Conceptualization.

## Declaration of competing interest

The authors declare no conflict of interest.

## References

[1] Alicka M., Marycz K. (2018). The effect of chronic inflammation and oxidative and endoplasmic reticulum stress in the course of metabolic syndrome and its therapy. Stem Cell. Int..

[2] Moszak M., Szulińska M., Bogdański P. (2020). You are what you eat-the relationship between diet, microbiota, and metabolic disorders-A review. Nutrients.

[3] Tasic N., Jakovljevic V.L.J., Mitrovic M., Djindjic B., Tasic D. (2021). Black chokeberry aronia melanocarpa extract reduces blood pressure, glycemia and lipid profile in patients with metabolic syndrome : a prospective controlled trial. Mol. Cell. Biochem..

[4] Niewiadomska J., Gajek-Marecka A., Gajek J., Noszczyk-Nowak A. (2022). Biological potential of polyphenols in the context of metabolic syndrome: an analysis of studies on animal models. Biology.

[5] Michicotl-Meneses M.M., Thompson-Bonilla M.d.R., Reyes-López C.A., García-Pérez B.E., López-Tenorio I.I., Ordaz-Pichardo C., Jaramillo-Flores M.E. (2021). Inflammation markers in adipose tissue and cardiovascular risk reduction by pomegranate juice in obesity induced by a hypercaloric diet in wistar rats. Nutrients.

[6] Kojadinovic M.I., Arsic A.C., Debeljak-Martacic J.D., Konic-Ristic A.I., Kardum N.D., Popovic T.B., Glibetic M.D. (2017). Consumption of pomegranate juice decreases blood lipid peroxidation and levels of arachidonic acid in women with metabolic syndrome. J. Sci. Food Agric..

[7] Pokimica B., García-Conesa M.T., Zec M., Debeljak-Martačić J., Ranković S., Vidović N., Petrović-Oggiano G., Konić-Ristić A., Glibetić M. (2019). Chokeberry juice containing polyphenols does not affect cholesterol or blood pressure but modifies the composition of plasma phospholipids fatty acids in individuals at cardiovascular risk. Nutrients.

[8] Petrovic S., Arsic A., Glibetic M., Cikiriz N., Jakovljevic V., Vucic V. (2016). The effects of polyphenol-rich chokeberry juice on fatty acid profiles and lipid peroxidation of active handball players: results from a randomized, double-blind, placebo-controlled study. Can. J. Physiol. Pharmacol..

[9] Kojadinovic M., Glibetic M., Vucic V., Popovic M., Vidovic N., Debeljak-Martacic J., Arsic A. (2021). Short-Term consumption of pomegranate juice alleviates some metabolic disturbances in overweight patients with dyslipidemia. J. Med. Food.

[10] Grabež M., Škrbić R., Stojiljković M.P., Rudić-Grujić V., Paunović M., Arsić A., Petrović S., Vučić V., Mirjanić-Azarić B., Šavikin K., Menkovic N., Jankovic T., Vasiljevic N. (2020). Beneficial effects of pomegranate peel extract on plasma lipid profile, fatty acids levels and blood pressure in patients with diabetes mellitus type-2: a randomized, double-blind, placebo-controlled study. J. Funct.Foods.

[11] Kim Y., Keogh J.B., Clifton P.M. (2016). Polyphenols and glycemic control. Nutrients.

[12] Behl T., Bungau S., Kumar K., Zengin G., Khan F., Kumar A., Kaur R., Venkatachalam T., Tit D.M., Vesa C.M., Barsan G., Mosteanu D.E. (2020). Pleotropic effects of polyphenols in cardiovascular system. Biomed. Pharmacother..

[13] Ashigai H., Komano Y., Wang G., Kawachi Y., Sunaga K., Yamamoto R., Takata R., Miyake M., Yanai T. (2018). Effect of administrating polysaccharide from black currant (*Ribes nigrum* L.) on atopic dermatitis in NC/Nga mice. Biosci. Microbiota Food Health.

[14] Karjalainen R., Anttonen M., Saviranta N. (2009). A review on bioactive compounds in black currants (Ribes nigrum L.) and their potential health-promoting properties. Acta Hortic..

[15] Tabart J., Franck T., Kevers C., Pincemail J., Serteyn D., Defraigne J., Dommes J. (2014). Antioxidant and anti-inflammatory activities of Ribes nigrum extracts. Food Chem..

[16] Trajković, M., Kitić, D., Mihajilov-Krstev, T., & Šavikin, K. Antimicrobial activity evaluation of black currant (*Ribes nigrum* L.) variety Čačanska crna juice and extract. Acta Fac. Med. Naissensis, 40, 208–216, doi:10.5937/afmnai40-41954.

[17] Branković S., Miladinović B., Radenković M., Gočmanac Ignjatović M., Kostić M., Šavikin K., Kitić D. (2016). Hypotensive, cardiodepressant, and vasorelaxant activities of black currant (Ribes nigrum 'Ben Sarek') juice. Can. J. Physiol. Pharmacol..

[18] Olejnik A., Kaczmarek M., Olkowicz M., Kowalska K., Juzwa W. (2018). ROS-modulating anticancer effects of gastrointestinally digested *Ribes nigrum* L. Fruit extract in human colon cancer cells. J. Funct.Foods.

[19] Alimbetov D., Jin Y.N., Gordon M.H., Lovegrove J.A. (2010). The effects of acute consumption of a blackcurrant juice drink on markers of endothelial function as a risk factor for CVD. Proc. Nutr. Soc..

[20] Bosnjakovic D., Ognjanov V., Ljubojevic M., Barac G., Predojevic M., Mladenovic E., Cukanovic J. (2012). Biodiversity of wild fruit species of Serbia. Genetika.

[21] Szczepaniak O.M., Kobus J., Weronika C., Monika K. (2019). Functional properties of cornelian cherry (Cornus mas L.): a comprehensive review. Eur. Food Res. Technol..

[22] Natić M., Pavlović A., Lo F., Nemanja B., Dragana S., Zagorac D. (2018). Nutraceutical properties and phytochemical characterization of wild Serbian fruits. Eur. Food Res. Technol..

[23] Zdunic G., Savikin K., Pljevljakusic D., Djordjevic B. (2016). Nutritional composition of fruit cultivars.

[24] Mohammadi K., Alizadeh Sani M., Nattagh-Eshtivani E., Yaribash S., Rahmani J., Shokrollahi Yancheshmeh B., Julian McClements D. (2021). A systematic review and meta-analysis of the impact of cornelian cherry consumption on blood lipid profiles. Food Sci. Nutr..

[25] Park J.H., Kho M.C., Kim H.Y., Ahn Y.M., Lee Y.J., Kang D.G., Lee H.S. (2015). Blackcurrant suppresses metabolic syndrome induced by high-fructose diet in rats. Evid. base Compl. Alternative Med..

[26] Paunovic M., Kotur-Stevuljevic J., Arsic A., Milosevic M., Todorovic V., Guzonjic A., Vucic V., Petrovic S. (2023). Antioxidative effects of black currant and cornelian cherry juices in different tissues of an experimental model of metabolic syndrome in rats. Antioxidants.

[27] Livak K.J., Schmittgen T.D. (2001). Analysis of relative gene expression data using real-time quantitative PCR and the 2(-Delta Delta C(T)) Method. Methods.

[28] Gholamrezayi A., Aryaeian N., Rimaz S., Abolghasemi J., Fallah S., Moradi N., Taghizadeh M. (2019). The effect of Cornus mas fruit extract consumption on lipid profile, glycemic indices, and leptin in postmenopausal women- A randomized clinical trial. Phytother Res..

[29] Grancieri M., Verediano T.A., Sant'Ana C.T., de Assis A., Toledo R.L., de Mejia E.G., Martino H.S.D. (2022). Digested protein from chia seed (Salvia hispanica L) prevents obesity and associated inflammation of adipose tissue in mice fed a high-fat diet. PharmaNutrition.

[30] Theodoro J.M.V., Grancieri M., Gomes M.J.C., Toledo R.C.L., de São José V.P.B., Mantovani H.C., Carvalho C.W.P., da Silva B.P., Martino H.S.D. (2022). Germinated millet (*Pennisetum glaucum* (L.) R. Br.) flour improved the gut function and its microbiota composition in rats fed with high-fat high-fructose diet. Int. J. Environ. Res. Publ. Health.

[31] Kim H.Y., Okubo T., Juneja L.R., Yokozawa T. (2010). The protective role of amla (Emblica officinalis Gaertn.) against fructose-induced metabolic syndrome in a rat model. Br. J. Nutr..

[32] Mishra N., Mohammed A., Rizvi S.I. (2017). Efficacy of Lepidium Sativum to act as an anti-diabetic agent. Prog. Health Sci..

[33] Ruxton C.H.S., Madeleine M. (2021). Fruit juices: are they helpful or harmful? An evidence review. Nutrients.

[34] Agarwal S., Fulgoni V.L., Welland D. (2019). Intake of 100% fruit juice is associated with improved diet quality of adults: NHANES 2013–2016 analysis. Nutrients.

[35] Osakabe N., Hoshi J., Kudo N., Shibata M. (2014). The flavan-3-ol fraction of cocoa powder suppressed changes associated with early-stage metabolic syndrome in high-fat diet-fed rats. Life Sci..

[36] Jakovljevic V., Milic P., Bradic J., Jeremic J., Zivkovic V., Srejovic I., Nikolic Turnic T., Milosavljevic I., Jeremic N., Bolevich S., Labudovic Borovic M., Mitrovic M., Vucic V. (2018). Standardized *Aronia melanocarpa* extract as novel supplement against metabolic syndrome: a rat model. Int. J. Mol. Sci..

[37] Luna-Vital D., Luzardo-Ocampo I., Cuellar-Nuñez M.L., Loarca-Piña G., Gonzalez de Mejia E. (2020). Maize extract rich in ferulic acid and anthocyanins prevents high-fat-induced obesity in mice by modulating SIRT1, AMPK and IL-6 associated metabolic and inflammatory pathways. J. Nutr. Biochem..

[38] Rodriguez-Ramiro I., Vauzour D., Minihane A.M. (2016). Polyphenols and non-alcoholic fatty liver disease: impact and mechanisms. Proc. Nutr. Soc..

[39] Feldman F., Koudoufio M., Desjardins Y., Spahis S., Delvin E., Levy E. (2021). Efficacy of polyphenols in the management of dyslipidemia: a focus on clinical studies. Nutrients.

[40] Tian J., Wu X., Zhang M., Zhou Z., Liu Y. (2018). Comparative study on the effects of apple peel polyphenols and apple flesh polyphenols on cardiovascular risk factors in mice. Clin. Exp. Hypertens..

[41] Rafiei H., Omidian K., Bandy B. (2019). Dietary polyphenols protect against oleic acid-induced steatosis in an in vitro model of NAFLD by modulating lipid metabolism and improving mitochondrial function. Nutrients.

[42] el Malik A., Sabahelkhier M.K. (2019). Changes in lipid profile and heart tissues of wistar rats induces by using monosodium glutamate as food additive. Int. J. Biochem. Physiol..

[43] Metabolism V.K. (1983). Comparative structures of rat and human plasma lipoproteins. Nutr. Rev..

[44] Raji M.B.L., Sathishkumar D.P.C. (2016). High-fructose diet is as detrimental as high-fat diet in the induction of insulin resistance and diabetes mediated by hepatic/pancreatic endoplasmic reticulum (ER) stress. Mol. Cell. Biochem..

[45] Lozano I., Van der Werf R., Bietiger W., Seyfritz E., Peronet C., Pinget M., Jeandidier N., Maillard E., Marchioni E., Sigrist S., Dal S. (2016). High-fructose and high-fat diet-induced disorders in rats: impact on diabetes risk, hepatic and vascular complications. Nutr. Metab..

[46] Elrazek A.M.A., Ibrahim S.R., El H.A. (2022). The ameliorative effect of Apium graveolens & curcumin against non - alcoholic fatty liver disease induced, by high fructose - high fat diet in rats. Future Journal of Pharmaceutical Sciences.

[47] Saravanan N., Patil M.A., Kumar P.U., Suryanarayana P., Reddy G.B. (2017). Dietary ginger improves glucose dysregulation in a long-term high-fat high-fructose fed prediabetic rat model. Indian J. Exp. Biol..

[48] Kim J.I., Huh J.Y., Sohn J.H., Choe S.S., Lee Y.S., Lim C.Y., Jo A., Park S.B., Han W., Kim J.B. (2015). Lipid-overloaded enlarged adipocytes provoke insulin resistance independent of inflammation. Mol. Cell Biol..

[49] Hardy O.T., Czech M.P., Corvera S. (2012). What causes the insulin resistance underlying obesity?. Curr. Opin. Endocrinol. Diabetes Obes..

[50] Williamson G., Sheedy K. (2020). Effects of polyphenols on insulin resistance. Nutrients.

[51] Aryaeian N., Sedehi S.K., Arablou T. (2017). Polyphenols and their effects on diabetes management: a review. Med. J. Islam. Repub. Iran.

[52] Serino A., Salazar G. (2018). Protective role of polyphenols against vascular inflammation, aging and cardiovascular disease. Nutrients.

[53] Klein A.V., Kiat H. (2015). The mechanisms underlying fructose-induced hypertension: a review. J. Hypertens..

[54] Bianchi F., Cappella A., Gagliano N., Sfondrini L., Stacchiotti A. (2022). Polyphenols-gut-heart: an impactful relationship to improve cardiovascular diseases. Antioxidants.

[55] Kousar M., Salma U., Khan T., Shah A.J. (2022). Antihypertensive potential of tartaric acid and exploration of underlying mechanistic pathways. Dose Response.

[56] Ristic-Medic D., Bajerska J., Vucic V. (2022). Crosstalk between dietary patterns, obesity and nonalcoholic fatty liver disease. World J. Gastroenterol..

[57] Salomone F., Godos J., Zelber-Sagi S. (2016). Natural antioxidants for non-alcoholic fatty liver disease: molecular targets and clinical perspectives. Liver Int..

[58] Tilg H., Moschen A.R. (2010). Evolution of inflammation in nonalcoholic fatty liver disease: the multiple parallel hits hypothesis. Hepatology.

[59] Lee J.S., Jun D.W., Kim E.K., Jeon H.J., Nam H.H., Saeed W.K. (2015). Histologic and metabolic derangement in high-fat, high-fructose, and combination diet animal models. TheScientificWorldJOURNAL.

[60] Abenavoli L., Larussa T., Corea A., Procopio A.C., Boccuto L., Dallio M., Federico A., Luzza F. (2021). Dietary polyphenols and non-alcoholic fatty liver disease. Nutrients.

[61] Bayram H.M., Majoo F.M., Ozturkcan A. (2021). Polyphenols in the prevention and treatment of non-alcoholic fatty liver disease: an update of preclinical and clinical studies. Clin. Nutr. ESPEN.

[62] Schmidt-Arras D., Rose-John S. (2016). IL-6 pathway in the liver: from physiopathology to therapy. J. Hepatol..

[63] Miller A.M., Wang H., Bertola A., Park O., Horiguchi N., Ki S.H., Yin S., Lafdil F., Gao B. (2011). Inflammation-associated interleukin-6/signal transducer and activator of transcription 3 activation ameliorates alcoholic and nonalcoholic fatty liver diseases in interleukin-10-deficient mice. Hepatology.

[64] Ueki K., Kondo T., Kahn C.R. (2004). Suppressor of cytokine signaling 1 (SOCS-1) and SOCS-3 cause insulin resistance through inhibition of tyrosine phosphorylation of insulin receptor substrate proteins by discrete mechanisms. Mol. Cell Biol..

[65] Grabež M., Škrbić R., Stojiljković M.P., Vučić V., Rudić Grujić V., Jakovljević V., Djuric D.M., Suručić R., Šavikin K., Bigović D., Vasiljević N. (2022). A prospective, randomized, double-blind, placebo-controlled trial of polyphenols on the outcomes of inflammatory factors and oxidative stress in patients with type 2 diabetes mellitus. Rev. Cardiovasc. Med..

[66] Papuc C., Goran G.V., Predescu C.N., Tudoreanu L., Ștefan G. (2022). Plant polyphenols mechanisms of action on insulin resistance and against the loss of pancreatic beta cells. Crit. Rev. Food Sci. Nutr..

[67] Marroquí L., Alonso-Magdalena P., Merino B., Fuentes E., Nadal A., Quesada I. (2014). Nutrient regulation of glucagon secretion: involvement in metabolism and diabetes. Nutr. Res. Rev..

