# Supplementary material for: Polyphenol-rich black currant and cornelian cherry juices ameliorate metabolic syndrome induced by a high-fat high-fructose diet in Wistar rats
Source: Heliyon. 2024 Mar 11;10(7):e27709. doi: 10.1016/j.heliyon.2024.e27709 (PMC10999883; doi:10.1016/j.heliyon.2024.e27709)
Supplement: Multimedia component 1 [file mmc1.docx]

**Table 1.** Composition of the diet fed to rats.

| Ingredients | Diet | | |  |
| --- | --- | --- | --- | --- |
|  | Standard chow diet (%) | | HFF diet (%) | |
| Proteins  Fats  Carbohydrate | 20  4.2  49.3 | 11  27.3  47.4 | | |
| Cellulose  Starch  Sugars  Moister  Ashes | 8  38  3.3  13  10 | 4.4  20.9  22.1  7.1  5.5 | | |
| Energy kJ/100g | 1100 | 1813 | | |
